# Immunomodulatory Effects of Taiwanese *Neolitsea* Species on Th1 and Th2 Functionality

**DOI:** 10.1155/2017/3529859

**Published:** 2017-07-11

**Authors:** Yin-Hua Cheng, Ying-Chi Lin, Ih-Sheng Chen, Sian-De Liu, Jih-Heng Li, Chia-Chi Wang

**Affiliations:** ^1^PhD Program in Toxicology, Kaohsiung Medical University, Kaohsiung 80708, Taiwan; ^2^School of Pharmacy, College of Pharmacy, Kaohsiung Medical University, Kaohsiung 80708, Taiwan; ^3^Department of Medical Research, Kaohsiung Medical University Hospital, Kaohsiung 80708, Taiwan

## Abstract

*Neolitsea* species, medicinal plants belonging to Lauraceae, contain rich alkaloids, steroids, sesquiterpenoids, and triterpenoids which possess antimicrobial, antioxidant, and anti-inflammatory bioactivities. However, species differences in the immunomodulatory effects and evidence pertaining to the effects of *Neolitsea* species on adaptive immunity are scarce. This study aimed to evaluate the immunomodulatory properties of ten Taiwanese *Neolitsea* plants on T helper (Th) cell functionality, especially Th1 and Th2. Most of the 29 crude extracts of *Neolitsea* were not toxic to splenocytes, except *N. buisanensis* roots. *N. aciculata* and *N. villosa* leaf extracts possessed differential immunomodulatory effects on Th1/Th2 balance. *N. aciculata* var. *variabillima* and *N. hiiranensis* leaf extracts attenuated both Th1 and Th2 cytokines while *N. konishii* dramatically suppressed IFN-*γ* production. As *N. aciculata* var. *variabillima* and *N. konishii* leaf extracts significantly attenuated Th1 functionality, we further evaluated their effects on CD4 cells under CD3/CD28 stimulation. *N. aciculata* var. *variabillima* significantly suppressed IFN-*γ*, IL-10, and IL-17, demonstrating the broad suppressive effects on T helper cells; *N. konishii* significantly suppressed IFN-*γ* and IL-10 production, while the production of IL-17 was not altered. Collectively, these data demonstrated that leaf extracts of Taiwanese *Neolitsea* species contain phytochemicals with potentials to be developed as selective immunomodulators.

## 1. Introduction

T cells play a pivotal role in the immune responses. They participate in a wide range of immune responses through a complicated cytokine network and via cell-cell interaction with other cells. Interleukin-2 (IL-2), a major autocrine and/or paracrine T-cell growth factor, is primarily produced by T helper cells and participates in the development and activation of T cells [1, 2]. T helper type 1 (Th1) cells produce interferon gamma (IFN-*γ*) to regulate immune responses and inflammation against viral and intracellular bacterial infections and inhibit tumor formation via the stimulation of antibody production and the activation of macrophage, cytotoxic T lymphocytes (CTL), and natural killer cells [3, 4]. On the other hand, Th2 cells induce interleukin-4 (IL-4) production to mediate the activation and maintenance of the humoral and/or allergy immune response against extracellular parasites, bacteria, allergens, and toxins [5]. The dysfunction of T cells, such as the imbalance of Th1/Th2 responses and abnormal immunostimulation, may lead to a variety of immune diseases. The excessive amounts of IFN-*γ* have been associated with several Th1-mediated immune disorders, such as delayed type hypersensitivity [6], Crohn's disease [7], and multiple sclerosis [8]. Immunosuppressants, developed for the treatment of these overreactive immune responses, also decrease normal immune responses and thereby increase the susceptibility of the patients to infections [9, 10]. Therefore, it is important to develop immunoregulators with less severe side effects. Natural compounds are under intensive investigation and showed promising progressions [11–13]. Several medicinal plants have been reported to regulate inflammatory responses in a variety of different animal models by attenuation of interleukin-2 (IL-2) and interferon-*γ* (IFN-*γ*) production [14–18].

*Neolitsea*, small evergreen trees or evergreen shrubs in the family Lauraceae, consists of about 100 species distributed in the tropics, especially in Brazil and Southern Eastern Asia. In Taiwan, there are 12 *Neolitsea* species and four of them, including *N. acuminatissima*, *N. daibuensis*, *N. hiiranensis*, and *N. parvigemma*, are endemic [19]. Recent studies on the diversity of phytochemical structures and bioactivities revealed the application potential of *Neolitsea* plants in industrial and medical fields [20, 21]. Parts of *Neolitsea* plants have been used in folk medicine for long periods of time in Asia. For example, the roots of *N. aurata* and the seeds of *N. chuii* are used to alleviate edema. The leaves of *N. cambodiana* are applied to treat furuncle and carbuncle. The roots of *N. zeylanica* have been shown to relieve rheumatic arthralgia [22]. Studies on the chemistry and pharmacology of *Neolitsea* species have led to the isolation and identification of more than 150 compounds including alkaloids, terpenoids, sterols, steroids and their derivatives, flavonoids, essential oils, and fatty acids with diverse activities [21, 23]. The essential oils of *N. pallens* exhibited antioxidant and antibacterial activities [24]. Alkaloids of *N. konishii* possessed vasoconstricting effects on rat aorta [25]. Sesquiterpenes of the *N. parvigemma* showed inhibitory effects on platelet aggregation [26]. Our previous study has shown that leaf extracts of *N. hiiranensis* and its derived terpenoids possessed immunomodulatory effects via regulation of IFN-*γ* production [27]. However, due to the complexity of compositions within *Neolitsea* species, it is still unclear how other Taiwanese *Neolitsea* species modulate the functionality of immune cells. The objective of this study aimed to examine the immunomodulatory effects of Taiwanese *Neolitsea* species on T-cell immunity.

To evaluate the immunomodulatory effects of Taiwanese *Neolitsea* species on T-cell immunity, we cultured and stimulated the mouse primary splenocytes with concanavalin A (ConA), a well-known T-cell mitogen, to stimulate cytokine production [28, 29]. Splenocytes are consist of antigen-presenting cells, B cells, and various type of T cells and have been widely used as primary immune cells for studying the functionality of T cells [30–33]. IL-2, IFN-*γ*, and IL-4 were evaluated to determine the effects of these plant extracts on Th1/Th2 functionality. In the present study, leaf extracts, including *N. aciculata* var. *variabillima*, *N. acuminatissima*, *N. hiiranensis*, *N. konishii*, and *N. villosa*, significantly inhibited IFN-*γ* production. These *Neolitsea* extracts showed potentials to be developed as new therapeutic immunomodulators. The chemical components and mechanisms of these medicinal plants to modulate Th1 functionality warrants further investigation.

## 2. Materials and Methods

### 2.1. Reagents and Chemicals

All reagents were purchased from Sigma (St. Louis, MO) unless otherwise stated. Enzyme-linked immunosorbent assay (ELISA) sets for cytokine measurement were purchased from BD Biosciences (San Diego, CA). Fetal bovine serum (FBS) and cell culture supplies were from Hyclone (Logan, UT).

### 2.2. Plants and Extraction

Twenty-nine crude extracts were prepared and extracted with cold MeOH at room temperature [34]. These plants used here were identified by one of the authors, Prof. Ih-Sheng Chen, and the voucher specimens were deposited in the Herbarium of the College of Pharmacy, Kaohsiung Medical University, Kaohsiung, Taiwan. Traditional usage and known possible bioactivities of these 29 *Neolitsea* extracts were shown in Table 1.

### 2.3. Animals

Male BALB/c mice (five weeks old) were obtained from BioLasco (Ilan, Taiwan). On arrival, mice were randomly transferred to plastic cages containing aspen bedding and quarantined for at least 1 week. Mice were housed in a temperature (22°C ± 2°C)-, humidity (50% ± 20%)-, and light (12 h light/dark cycle)-controlled environment. Food and water were supplied ad libitum. All animal experimental procedures and housing have been approved by the Institutional Animal Care and Use Committee (IACUC) of Kaohsiung Medical University, Taiwan (Protocol ID: 106014). The experimental mice were euthanized by carbon dioxide mixed with oxygen for anesthesia with approved IACUC protocol and regulations.

### 2.4. Splenocyte Isolation

The mice were sacrificed, and their spleens were harvested and made into single-cell suspensions. The isolation of splenocytes was according to the previous procedures which have been described in detail before [35, 36]. Briefly, spleens were gently dissociated by teasing on a sterile 60-mesh steel screen (Sigma-Aldrich). The cell suspensions were washed in incomplete RPMI 1640 medium (Hyclone, Logan, UT) supplemented with 5% fetal bovine serum (Gibco, USA) and 1% penicillin and streptomycin (Amresco). Lymphocytes were enriched by removing red blood cells from splenocytes after treating with ACK lysis buffer. To activate splenocytes, cells were seeded in 48-well plates and stimulated with 5 *μ*g/mL of ConA (Sigma-Aldrich, St. Louis, MO). The splenocytes (5 × 10^6^ cells/mL) were either left untreated (control group) or exposed to crude extracts of *Neolitsea* extracts followed by stimulation with ConA (5 *μ*g/mL) for 48 h. The supernatants of culture wells were collected to detect the cytokine levels by enzyme-linked immunosorbent assay (ELISA).

### 2.5. Cell Viability

Splenocytes (5 × 10^6^ cells/mL) were seeded into 96-well plates. The cells were either left untreated or treated with crude extracts followed by stimulation with ConA for 48 h. The viability was determined by the 3-(4,5-dimethylthiazol-2-yl)-2,5-diphenyl-tetrazolium bromide (MTT) assay. A methyl-thiazol-tetrazolium stock solution (5 mg/mL in phosphate-buffered saline) was then added to each well (10 *μ*L/well) and incubated for 4 h. The formed formazan was dissolved in a lysis buffer (10% SDS in *N,N*-dimethylformamide) overnight in the dark. The optical density was measured at 570 nm (and at 630 nm as a background reference) using a microplate reader (Dynatech Laboratories Inc., Chantilly, VA).

### 2.6. Isolation of CD4 T Helper Cells

Mouse CD4 T helper cells were purified from splenocytes by using magnetic cell separation with the CD4 T Lymphocyte Enrichment Kit (BD Biosciences). To induce the polarization of Th cells, CD4 T cells (5 × 10^5^ cells/mL) were seeded in the 48-well flat bottom tissue culture plates which were precoated with anti-mouse CD3 antibody (1 *μ*g/mL) overnight, and the cells were either left untreated or treated with *Neolitsea* extracts followed by stimulation with soluble anti-mouse CD28 antibody (1 *μ*g/mL) for 48 h. The culture supernatants and the cells were collected for ELISA assay and intracellular cytokine staining, respectively.

### 2.7. Measurement of Th1/Th2 Cytokines of Total T Cells and CD3/CD28-Stimulated CD4 T Cells by Enzyme-Linked Immunosorbent Assay (ELISA)

To examine the immunomodulatory activities of *N.* species on the total T cells, the splenocytes (5 × 10^6^ cells/mL) and CD4 T cells were cultured in 48-well plates (300 *μ*L/well) and either left untreated or treated with crude extracts followed by stimulation with ConA (5 *μ*g/mL) or CD3/CD28 stimulation for 48 h. The supernatants were harvested and quantified for IL-2, IFN-*γ*, IL-4, IL-10, and IL-17 by sandwich ELISA kits according to the manufacturer's instructions (BD Biosciences).

### 2.8. Intracellular Cytokine Staining by Flow Cytometry Analysis

CD4 T cells were cultured in a 48-well plate and treated with *N.* species for 36 h. For analysis of intracellular cytokine production, the cells then treated with GolgiStop (0.6 mL/mL; BD Biosciences) for 10 h prior to being harvested for antibody staining. The CD4 T cells then were fixed and permeabilized using Fixation and Perm/Wash buffers (BD Biosciences) before intracellular IFN-*γ* and IL-4 staining by PE-conjugated anti-mouse IFN-*γ* and IL-4 mAb (clone XMG1.2 and 11B11; Biolegend). Ten thousand of CD4 T cells were acquired on a BD LSR II flow cytometer (BD Biosciences). The mean fluorescence intensity (MFI) of IFN-*γ* in total CD4 T cells was quantified by gating CD4 T cells and then analyzed using FlowJo software (Treestar, Inc., CA).

### 2.9. Statistical Analysis

All the data were analyzed using a GraphPad software Prism 5.0 (GraphPad Software Inc., San Diego, CA, USA). Each treatment group was measured in quadruplicate and the data were presented as the mean ± standard error (SE). Data were analyzed using a one-way analysis of variance (ANOVA) for multiple comparisons and Dunnett's two-tailed *t-*test was used to compare the results for the treatment groups with vehicle control group. *p* < 0.05 was defined as statistically significant.

## 3. Results

### 3.1. The Effects of Crude Extracts of *Neolitsea (N.)* Species on Cell Viability and IL-2 Production by Murine Primary Splenocytes In Vitro

**Figure 1:**
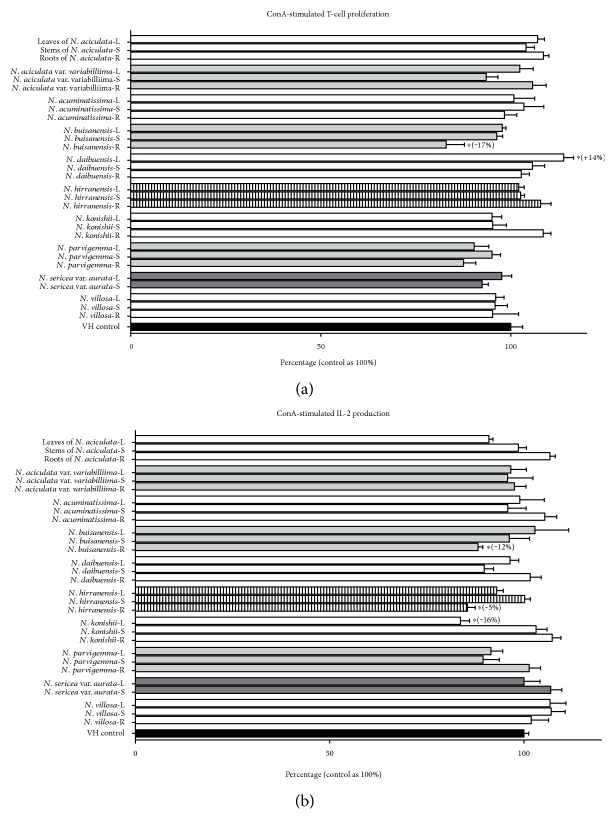
The effects of Taiwanese *Neolitsea* plants on T-cell viability and activation. Splenocytes (5 × 10^6^) were treated with vehicle control (VH, 0.05% DMSO) and/or various crude extracts of *Neolitsea* plants (10 *μ*g/mL) followed by ConA treatment for 48 h. (a) The cell viability was determined using an MTT assay and the level of (b) IL-2 in the supernatants was quantified by ELISA. The viability and IL-2 level of the VH-treated group was shown as 100%. L, S, and R were represented as leaf, stem, and root parts of different plants. The effects of *N. aciculata*, *N. aciculata* var. *variabillima*, *N. acuminatissima*, *N. buisanensis*, *N. daibuensis*, *N. hiiranensis*, *N. konishii*, *N. parvigemma*, *N. sericea* var. *aurata*, and *N. villosa* on (a) cell viability and (b) IL-2 production were shown from top to bottom, respectively. Data were expressed as the mean ± SE of triplicated cultures. Results were pooled from three independent experiments. ^∗^*p* < 0.05, compared to the VH group.

To evaluate the effects of *N.* species on splenocyte viability, we first investigated the direct cytotoxicity of *N.* species in vitro. Most of these crude extracts at the concentration of 10 *μ*g/mL did not significantly affect the cell viability compared to vehicle control (VH was referred as 100%), except for the roots of the *N. buisanensis* which reduced the cell viability by 17% (Figure 1(a) and Supplemental Table 1 available online at https://doi.org/10.1155/2017/3529859). As IL-2 plays important roles in T-cell clonal expansion and activation, the supernatants of the treated groups were collected to determine the IL-2 levels. Most of the crude extracts of *N.* species did not alter IL-2 production, except for the roots of the *N. hiiranensis* and the leaves of the *N. konishii* which suppressed IL-2 production by 15% and 16% (at the concentration of 10 *μ*g/mL), respectively (Figure 1(b) and Supplemental Table 1). Collectively, these data showed that the root part of *N. buisanensis* is relatively toxic to primary immune cells compared to other extracts. It should be circumspectly considered for further development of the immunomodulatory ingredients from this plant. Moreover, the roots of the *N. hiiranensis* and the leaves of the *N. konishii* suppressed IL-2, revealing that these extracts may affect the maturation and early activation of T helper cells.

### 3.2. *Neolitsea* Crude Extracts Differentially Modulated Th1 and Th2 Cytokines Production

**Figure 2:**
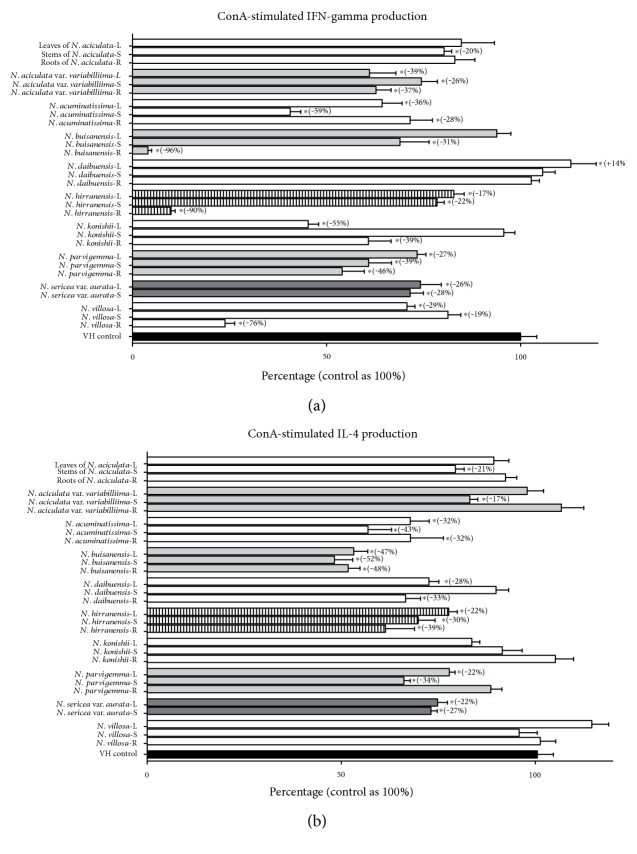
The effects of Taiwanese *Neolitsea* plants on Th1/Th2 cytokine production. Splenocytes (5 × 10^6^) were treated with vehicle control (VH, 0.05% DMSO) and/or various crude extracts of *Neolitsea* plants (10 *μ*g/mL) followed by ConA stimulation for 48 h. The supernatants were collected for measuring the concentration of cytokines by ELISA. The cytokine level of the VH-treated group was shown as 100%. L, S, and R were represented as leaf, stem, and root parts of different plants. The effects of *N. aciculata*, *N. aciculata* var. *variabillima*, *N. acuminatissima*, *N. buisanensis*, *N. daibuensis*, *N. hiiranensis*, *N. konishii*, *N. parvigemma*, *N. sericea* var. *aurata*, and *N. villosa* on (a) IFN-*γ* and (b) IL-4 were shown from top to bottom, respectively. Data were expressed as the mean ± SE of triplicated cultures. Results were pooled from three independent experiments. ^∗^*p* < 0.05, compared to the VH group.

We next investigated the effects of crude extracts of *N.* species on ConA-stimulated cytokine production by splenocytes (Figure 2 and Supplemental Table 1). IFN-*γ* and IL-4, which belong to the Th1 and Th2 signature cytokines, were determined to study the effects of plant-derived extracts (10 *μ*g/mL) on Th1/Th2 immune responses. *N. aciculata* did not affect the production of IFN-*γ* and IL-4 except that the stem extracts of *N. aciculata* inhibited both IFN-*γ* and IL-4 by approximately 20% compared to VH control. These data suggested that the stem of *N. aciculata* inhibited both Th1 and Th2 functionality. The leaves and roots of *N. aciculata* var. *variabillima* (gray bar) significantly suppressed IFN-*γ* production by 37–39%, while the IL-4 was not altered. Interestingly, the stem of *N. aciculata* var. *variabillima* not only inhibited IFN-*γ* (26%) but also inhibited IL-4 (17%). These data indicated that Th1 cells were more sensitive to be suppressed by components of *N. aciculata* var*. variabillima*, and the leaf and root parts differentially modulated Th1 functionality. Different parts of *N. acuminatissima* (white bar) inhibited both IFN-*γ* and IL-4 cytokine productions by 28–59% and 32–43%, respectively. It was speculated that *N. acuminatissima* may affect the development of total Th cells in response to ConA stimulation. The leaves of *N. buisanensis* (gray bar) did not affect the IFN-*γ* but reduced IL-4 indicating the differential modulatory effects on Th2 functionality. *N. hiiranensis* attenuated IFN-*γ* by 17%–90% and inhibited IL-4 by 22–39%. It is suggested that the whole extract of *N. hiiranensis* modulated both Th1 and Th2 activities (slash gray bar). The leaves and roots of *N. konishii* mainly attenuated IFN-*γ* by 39–55% while IL-4 was not affected. The leaf and stem parts of *N. parvigemma* inhibited Th1 and Th2 cytokine production by 22–39% (gray bar). The leaves and stems of *N. sericea* var. *aurata* inhibited both IFN-*γ* and IL-4 (22–28% inhibition rate). Finally, *N. villosa* extracts attenuated IFN-*γ* by 17–76%, but IL-4 was not altered. Taken together, the above results showed that crude extracts of *N.* species differentially modulated T helper cell functionality. The leaf extracts of *N. acuminatissima*, *N. hiiranensis*, and *N. parvigemma* and *N. sericea* var. *aurata* suppressed both Th1 and Th2 functionality. *N. aciculata* var. *variabillima*, *N. konishii*, and *N. villosa* mainly inhibited IFN-*γ* production. In contrast, *N. buisanensis* and *N. daibuensis* attenuated IL-4 production.

### 3.3. The Differential Immunomodulatory Effects of Leaf and Root Extracts of Selected *Neolitsea* Species on Th1/Th2 Functionality in Dose-Dependent Manners

According to our abovementioned results, several leaf extracts of *Neolitsea* species may differentially regulate the functionality of Th1 and Th2 cells by modulating the production of IFN-*γ* and IL-4 cytokines. We next further evaluated the concentration-dependent effects of these particular leaf extracts on ConA-stimulated splenocytes. The effects of leaf extracts of *N. aciculata*, *N. aciculata* var. *variabillima*, *N. daibuensis*, *N. hiiranensis*, *N. konishii*, and *N. villosa* on the cell viability and Th1/Th2 cytokine secretions were shown in Table 2. *N. aciculata* at the concentration of 5–50 *μ*g/mL did not alter the viability as well as IL-4 production. In contrast, *N. aciculata* concentration dependently inhibited IFN-*γ* (25–50 *μ*g/mL) while IL-2 was suppressed at the concentration of 50 *μ*g/mL, indicating that leaf extracts of *N. aciculata* mainly affect Th1 functionality from 25 *μ*g/mL (Table 2). *N. aciculata* var. *variabillima* (5–50 *μ*g/mL) dramatically attenuated IFN-*γ* production while IL-2 was suppressed at the concentration of 25–50 *μ*g/mL. *N. aciculata* var. *variabillima* (50 *μ*g/mL) significantly inhibited cell viability and all tested cytokines demonstrating the cytotoxic effects of this plant at high concentration (Table 2). *N. daibuensis* (10–25 *μ*g/mL) attenuated IL-4 production while Th1 cytokines were not altered, demonstrating its differential effects on Th2 functionality (Table 2). *N. hiiranensis* did not affect cell viability and IL-2 production. Interestingly, both Th1 and Th2 cytokines were attenuated by *N. hiiranensis* leaf extracts (Table 2). *N. konishii* dramatically suppressed IFN-*γ* at the concentration of 5–25 *μ*g/mL while the cell viability was not altered. In addition, IL-2 production was suppressed from 5 to 25 *μ*g/mL. These data indicated the potential effects of *N. konishii* on Th1 functionality (Table 2). Interestingly, *N. villosa* at the concentration of 25 *μ*g/mL slightly increased cell viability and IL-4 production while IFN-*γ* was significantly suppressed. This data demonstrated the differential modulatory effects of *N. villosa* on Th1/Th2 balance (Table 2). As the leaf extracts of *N. aciculata* var. *variabillima* and *N. konishii* mainly inhibited IFN-*γ* production, we further determined the effects of leaf extracts on IL-12 production and their root extracts on the functionality of T cells. The leaves of *N. aciculata* var. *variabillima* did not affect IL-12 production; however, *N. konishii* dramatically attenuated IL-12 secretion at 25 *μ*g/mL, revealing that leaf extracts of *N. konishii* suppressed IFN-*γ* production through the downregulation of upstream IL-12 production by splenic dendritic cells (Supplemental Fig.1). The root extracts of *N. aciculata* var. *variabillima* attenuated IL-2 and IFN-*γ* production. The root extracts of *N. konishii* dramatically suppressed IFN-*γ* at the concentration of 5–50 *μ*g/mL. These results demonstrated that both *N. aciculata* var. *variabillima* and *N. konishii* significantly suppressed Th1 functionality (Table 3).

### 3.4. The Direct Immunomodulatory Effects of Leaf Extracts of Selected *Neolitsea* Species on Th1/Th2 Functionality

**Figure 3:**
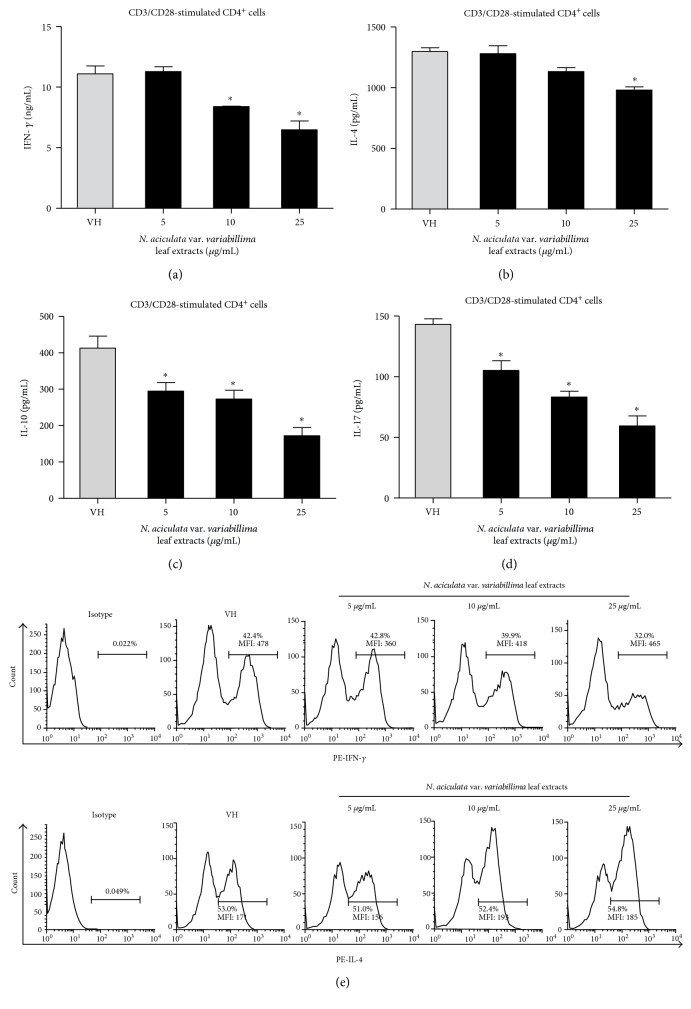
The effects of leaf extracts of *N. aciculata* var. *variabillima* on T-cell functionality. The enriched CD4 T cells (5 × 10^5^ cells/mL) were stimulated with anti-CD3 and anti-CD28 (1 *μ*g/mL) in the absence or in the presence of the leaf extracts of *N. aciculata* var. *variabillima* (5–25 *μ*g/mL) for 48 h. (a)–(d) The concentration of IFN-*γ*, IL-10, IL-4, and IL-17 in the supernatants was measured by ELISA. Data were expressed as the mean ± SE of quadruplicate cultures. ^∗^*p* < 0.05 was significant compared to the VH group. (e) The representative histogram of intracellular cytokine staining. Total percentage and the level of mean fluorescence intensity (MFI) of IFN-*γ*^+^ and IL-4^+^ cells in CD4^+^ T cells were shown. Results were representative of two independent experiments.

**Figure 4:**
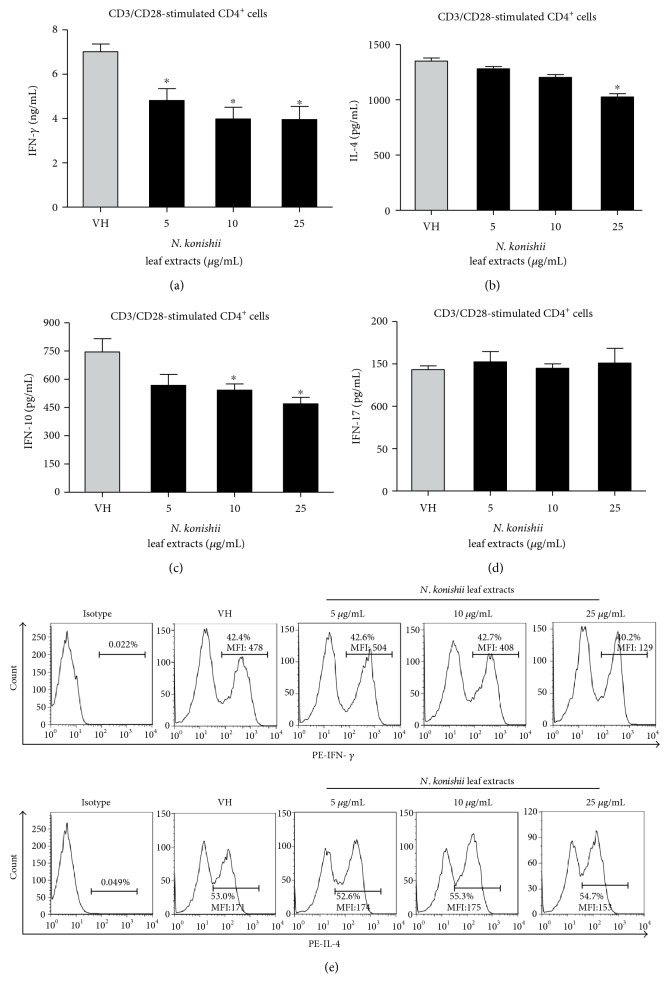
The effects of leaf extracts of *N. konishii* on T-cell functionality. The enriched CD4 T cells (5 × 10^5^ cells/mL) were stimulated with anti-CD3 and anti-CD28 (1 *μ*g/mL) in the absence or the presence of the leaf extracts of *N. konishii* (5–25 *μ*g/mL) for 48 h. (a)–(d) The concentration of IFN-*γ*, IL-10, IL-4, and IL-17 in the supernatants was measured by ELISA. Data were expressed as the mean ± SE of quadruplicate cultures. ^∗^*p* < 0.05 was significant compared to the VH group. (e) The representative histogram of intracellular cytokine staining. Total percentage and the level of mean fluorescence intensity (MFI) of IFN-*γ*^+^ and IL-4^+^ cells in CD4^+^ T cells were shown. Results were representative of two independent experiments.

Based on the above results, *N. aciculata* var. *variabillima* and *N. konishii* mainly modulated Th1 functionality in ConA-stimulated splenocytes. We next analyzed the direct immunomodulatory effects of these two leaf extracts on CD4 T cells under stimulation of CD3 and CD28. CD3 is a major component of the T-cell receptor (TCR) complex, as well as CD28, which is a costimulatory molecule. The activation of both CD3 and CD28 will induce T-cell proliferation and cytokine production [37]. In addition, the production of IL-10 and IL-17 were determined to study the effects of these leaf extracts on the functionality of regulatory T cells and Th17 cells. The leaves extracts of *N. aciculata* var. *variabillima* significantly suppressed IFN-*γ*, IL-10, and IL-17 production at the concentration of 5–25 *μ*g/mL in CD3/CD28 stimulated CD4 T cells (Figures 3(a), 3(c), and 3(d)), while IL-4 were slightly altered at the concentration of 25 *μ*g/mL (Figure 3(b)). The leaf extracts of *N. konishii* mainly attenuated IFN-*γ* and IL-10 productions at the concentration of 5–25 *μ*g/mL (Figure 4()), while IL-17 was not affected (Figure 4()). We also performed the intracellular staining to detect the protein levels of IFN-*γ* and IL-4 in CD4 T cells. Interesting, the leaf extracts of *N. aciculata* var. *variabillima* decreased the percentage of IFN-*γ*^+^ in CD4 T cells from 42% (VH) to 32%, but the level of mean fluorescence intensity of IFN-*γ* was not significantly altered in IFN-*γ*^+^ cells. *N. aciculata* var. *variabillima* did not affect the percentage of IL-4^+^ cells in CD4 T cells nor the protein levels of IL-4 in CD4 T cells, suggesting that the leaves of *N. aciculata* var. *variabillima* decreased the proportion of IFN-*γ^+^* in CD4 T cells. By contrast, the leaf extracts of *N. konishii* attenuated the protein level of IFN-*γ* in CD4 T cells while the proportion of IFN-*γ*^+^ cells were not changed.

## 4. Discussion

Natural products isolated from traditional medicinal plants have therapeutic effects in the prevention and treatment of various immune disorders. *Neolitsea* species exhibit extensive bioactivities and have been used as traditional herbal medicines in oriental countries. However, a comparative study of immunomodulatory properties of different *Neolitsea* species on immunocompetent cells such as T helper cells has not been demonstrated to date. Our previous reported study found that the leaf extracts of *N. hiiranensis* significantly inhibited IL-12, IFN-*γ*, and IL-2 cytokine productions as well as the serum levels of OVA-primed antigen-specific IgM and IgG_2a_ in vivo [27]. In the present study, we evaluated the immunomodulatory properties of ten Taiwanese *Neolitsea* plants on T helper cells. Our results showed that most crude extracts of Taiwanese *Neolitsea* species decreased IFN-*γ* production at concentrations below the IC_50_ by mitogen-stimulated splenocytes, and the immunomodulatory activities of *Neolitsea* extracts, especially leaf extracts, were mainly on the suppression of Th1 immunity.

Several reports showed that *Neolitsea* species and its derived secondary compounds, including sesquiterpenoids, triterpenoids, alkaloids, and steroids, possess several bioactivities including anti-inflammatory activities [18, 20]. *Neolitsea aciculata* essential oil (NAE) attenuated the *Propionibacterium acnes*-induced secretion of tumor necrosis factor-alpha (TNF-alpha) and interleukin-8 (IL-8) productions in human cell lines, revealing its anti-inflammatory effects [38]. Six furanogermacrane sesquiterpenes isolated from the stems of *N. parvigemma*, including deacetylzeylanidine, linderalactone, neolitrane, pseudoneolinderane, zeylanidine, and zeylanicine, have been shown to possess anti-inflammatory activities; among them, pseudoneolinderane and linderalactone have the ability to inhibit the superoxide anion generation by human neutrophils in response to formyl-l-methionyl-l-leucyl-l-phenylalanine/cytochalasin B (fMLP/CB) with IC_50_ values of 3.21 and 8.48 *μ*g/mL, respectively [39]. Daibucarboline A, isolinderalactone, 7-omethylnaringenin, and prunetin isolated from the roots of *N. daibuensis* exhibited moderate inhibition of inducible nitric oxide synthase (iNOS) [40]. Hiiranlactone B and hiiranlactone D, isolated from the leaves of *N. hiiranensis* were classified as sesquiterpenes and possessed anti-inflammatory and immunomodulatory effects [27, 34]. Thaliporphine, the alkaloid of the bark extracts of *N. konishii*, demonstrated vasoconstricting effects by promoting Ca^2+^ entry [25] and possessed antioxidant, anti-inflammatory, and antiapoptotic activity to prevent cardiovascular system disorder in guinea pigs [41]. In addition to the anti-inflammatory effects, the ingredients from *Neolitsea* species possess several bioactivities. Flavone glycosides, 2′-*p*-coumaroylafzelin and 2′,3′-di-*O*-(*p*-coumaroyl)afzelin, have been isolated from *N. aciculata* to demonstrate the antimelanogenesis activities [42]. Neolitacumone A–C and 2,6-dimethoxy-*p-*benzoquinone from the stem barks of *N. acuminatissima* displayed significant inhibitory activities against Hep 2.2.15 cells [43]. Isolinderalactone, a sesquiterpenes from the roots of *N. villosa*, exhibited antitumor activity [44]. Our recent study reported that *N. hiiranensis*-derived caryophyllene oxide inhibited several aspects of adaptive immune responses, including T-cell differentiation, IFN-*γ* production, and Th1-associated genes [27]. These studies suggested that the immunomodulatory effects of *Neolitsea* plants are worth further investigation; in addition, the present study aimed to clear how other Taiwanese *Neolitsea* species modulate the functionality of Th cells by ConA-stimulated splenocytes.

Th cells play pivotal roles in the acquired immunity. They are categorized into several subsets based on the cytokine production after stimulation [5]. The main roles of the type 1 Th cells (Th1) are against intracellular bacteria and protozoa. They are predominantly induced by interleukin-12 (IL-12) and are fully activated by IL-2 and IFN-*γ*. Induction of mitotic activity and cytokine production are associated with Th cell functionality. IFN-*γ*, an upstream cytokine of Th1 cells, regulates inflammatory immune response, promotes Th1 cell differentiation, enhances MHC class II expression on antigen-presenting cells, and possesses protective immune responses against cancer formation [4, 45–48]. Saha et al. reported that *Viscum album-*derived Qu Spez significantly stimulated IFN-*γ* secretion showing its ability to modulate the immune system and to suppress tumor regression by regulation of dendritic cells [49]. Th1 cells secreted IFN-*γ* to activate cell-mediated immune responses that several immune cells are involved such as macrophages, cytotoxic T cells, and natural killer cells [4, 45, 46, 50]. Th2 cells are against extracellular multicellular parasites by releasing interleukin-4 (IL-4), IL-5, and IL-13 cytokines [51]. Furthermore, the interaction of dendritic cells with activated B cells may help dendritic cells to acquire unique abilities to promote polarization of Th2 [50]. The imbalance of Th1/Th2 may lead to immunological diseases, such as rheumatoid arthritis, type-1 diabetes, multiple sclerosis, and asthma [52]. Discovering the selective immunomodulators on different subsets of Th cells may be beneficial for the treatment of immune disorders.

In our presented data, most of the leaf extracts of *Neolitsea* species have low cytotoxicity. The leaf extracts of *N. aciculata* and *N. villosa* possessed differential immunomodulatory effects on Th1/Th2 balance. *N. daibuensis* leaf extracts slightly attenuated IL-4 production; in contrast, *N. aciculata* var. *variabillima* and *N. hiiranensis* leaf extracts attenuated both Th1 and Th2 cytokines. *N. konishii* leaf extracts dramatically suppressed IFN-*γ* cytokine. The leaf extracts of *N. aciculata* var. *variabillima* and *N. konishii* differentially affect the functionality of different subsets of Th cells. Collectively, these *Neolitsea* species demonstrated selective immunomodulatory effects and the underlying mechanisms are needed for further study. Although there are a variety of immunosuppressive drugs such as cyclosporine A, tacrolimus, daclizumab, and basiliximab to prevent the rejection of transplanted organs and tissues, autoimmune diseases, and inflammatory disorders [53], these drugs could cause systemic immunosuppression which greatly increases the risks of tumor formation and infections [54]. Hence, development of new therapeutic and preventive immunomodulators from medicinal plants to manage immune disorders is of great importance. There are many studies devoted to discover new immunomodulatory therapeutic compounds from natural plants with low toxicity [55–57]. *p*-Coumaryl alcohol-gamma-*O*-methyl ether (CAME) isolated from *Alpinia galanga* was selectively and substantially suppressed in IFN-*γ* production in Th cells [11]. Farrerol, a new type of 2,3-dihydro-flavonoid isolated from the leaves of *Rhododendron dauricum* L, markedly suppressed concanavalin A- induced lymphocyte proliferation, Th1 and Th2 cytokine production, and differentiation of T helper cell populations [46]. Physalin H isolated from *Physalis angulata* exhibited an immunosuppressive activity on T-cell activation and proliferation by modulation of Th1/Th2 immune balance [58]. There are 14 compounds from leaves of *N. hiiranensis* that exhibited anti-inflammatory activity to suppress the generation of superoxide anion from neutrophils [34]. Furthermore, four major metabolites of the leaves of *N. hiiranensis*, including elemane type, caryophyllene type, aromadendrene type, eudesmane type, and germacrane dilactone type, are the main effective anti-inflammatory constituents [34]. Caryophyllene oxide, a sesquiterpene compound from the leaves of *N. hiiranensis*, inhibited the influx of neutrophil into the inflammatory site and the activation of NF-*κ*B pathway [59]. We recently reported that *N. hiiranensis*-derived terpenoids, including hiiranlactone D, *trans*-phytol, and *β*-caryophyllene oxide, attenuated antigen-specific T helper 1 immunity [27]. Here, the leaf extracts of *Neolitsea* plants showed potential immunomodulatory activities on T-cell functionality. To further study the potential, immunomodulatory compounds from these plants will help to discover new immunomodulators.

## 5. Conclusions

The study demonstrated that most of the crude extracts of Taiwanese *Neolitsea* species, especially leaf extracts, were not toxic to primary splenocytes, but they are capable of decreasing IFN-*γ* production without affecting IL-2 production by T cells. The selective Th1 immunomodulatory effects of the *Neolitsea* extracts indicate that the phytochemicals in these extracts have potential to be further evaluated and developed as immunomodulatory agents.

## Supplementary Material

The information of supplementary materials are as follows: Supplemental Table 1. The Effects of N. species extracts on Th1/Th2 cytokine production by ConA-stimulated splenocytes. Supplemental Fig 1. The effects of selected Neolitsea species extracts on IL-12 secretions by ConA-stimulated splenocytes. ConA-stimulated splenocytes (5∗106cells/mL) were either left untreated (NA) or re-stimulated with ConA (5 µg/mL) in the absence or the presence of selected Neolitsea species (5-50 µg/mL) for 48 h. (A-C) The levels of IL-12 secretions in the supernatants were quantified by ELISA assay. Data were expressed as the mean ± SE of quadruplicate cultures. Results were representative of two independent experiments. ∗p<0.05 was significant compared to the VH group.

## Figures and Tables

**Table 1:** The possible bioactivities of Taiwanese *Neolitsea* plants used in this study.

Scientific name	Parts of plant used	Known possible bioactivities
*N. aciculata*	Leaf, stem, and root	Antibacterial, antioxidant, and anti-inflammatory activities [38]
*N. aciculata* var. *variabillima*	Leaf, stem, and root	Need to be studied
*N. acuminatissima* ^∗^	Leaf, stem, and root	Cytotoxicity [43]
*N. buisanensis*	Leaf, stem, and root	Need to be studied
*N. daibuensis* ^∗^	Leaf, stem, and root	Anti-inflammation [40]
*N. hiiranensis* ^∗^	Leaf, stem, and root	Anti-inflammatory, antimicrobial [34], and immunomodulatory activities [27]
*N. konishii*	Leaf, stem, and root	Vasoconstriction [25], cardiotonic, and anti-inflammatory effects [41, 60]
*N. parvigemma* ^∗^	Leaf, stem, and root	Anti-inflammatory [39], antifungal [61], and antiplatelet activity [26]
*N. sericea* var. *aurata*	Leaf and stem	Antiradical activity [62]
*N. villosa*	Leaf, stem, and root	Cytotoxicity [44]

^∗^Taiwanese endemic *Neolitsea* species.

**Table 2:** The effects of *N.* species leaf extracts on Th1/Th2 cytokine production by ConA-stimulated splenocytes.

Plants	Concentration	Cell viability	Th1 cytokines		Th2 cytokines
	(*μ*g/mL)	MTT (O.D.)	IL-2 (ng/mL)	IFN-*γ* (ng/mL)	IL-4 (pg/mL)
	Control	0.17 ± 0.01	0.06 ± 0.04	Not detectable	Not detectable
*N. aciculata*	VH	0.52 ± 0.05	7.61 ± 0.42	119.30 ± 6.28	173.70 ± 8.07
	5	0.57 ± 0.04	7.23 ± 0.47	116.40 ± 0.91	174.10 ± 9.39
	10	0.59 ± 0.04	6.93 ± 0.39	121.30 ± 6.50	167.40 ± 8.63
	25	0.52 ± 0.04	6.40 ± 0.40	60.71 ± 6.23^∗^	195.50 ± 19.73
	50	0.41 ± 0.05	5.42 ± 0.27^∗^	42.15 ± 1.55^∗^	210.80 ± 16.73
*N. aciculata* var. *variabillima*	VH	0.39 ± 0.02	10.86 ± 0.90	91.71 ± 2.51	222.00 ± 33.97
	5	0.44 ± 0.02	10.34 ± 0.95	74.44 ± 6.36^∗^	246.20 ± 19.19
	10	0.40 ± 0.03	9.74 ± 0.86	62.35 ± 6.32^∗^	208.40 ± 19.92
	25	0.34 ± 0.01	7.45 ± 0.35^∗^	43.94 ± 3.21^∗^	161.80 ± 17.45
	50	0.27 ± 0.03^∗^	3.44 ± 0.52^∗^	10.58 ± 3.38^∗^	74.62 ± 9.46^∗^
*N. acuminatissima*	VH	0.64 ± 0.03	15.53 ± 0.30	98.21 ± 2.68	50.87 ± 4.75
	5	0.67 ± 0.03	15.31 ± 0.35	90.15 ± 2.80	40.28 ± 1.20^∗^
	10	0.67 ± 0.03	14.46 ± 0.54	41.33 ± 1.52^∗^	28.12 ± 2.90^∗^
	25	0.71 ± 0.02	11.80 ± 1.17^∗^	39.61 ± 3.91^∗^	19.61 ± 4.20^∗^
	50	0.62 ± 0.02	9.08 ± 1.75^∗^	22.45 ± 0.44^∗^	12.63 ± 1.98^∗^
*N. buisanensis*	VH	0.67 ± 0.02	19.22 ± 0.88	444.20 ± 10.79	44.90 ± 1.89
	5	0.67 ± 0.03	21.74 ± 2.60	407.70 ± 23.01	55.90 ± 9.88
	10	0.65 ± 0.02	21.05 ± 2.20	394.70 ± 16.88	21.40 ± 2.00^∗^
	25	0.64 ± 0.03	20.88 ± 2.55	339.70 ± 27.08^∗^	21.20 ± 6.40^∗^
	50	0.64 ± 0.02	18.87 ± 2.68	294.00 ± 5.38^∗^	11.40 ± 3.16^∗^
*N. daibuensis*	VH	0.60 ± 0.02	8.60 ± 0.18	157.30 ± 10.25	92.32 ± 5.13
	5	0.63 ± 0.01	8.20 ± 0.23	179.20 ± 7.68	83.36 ± 3.57
	10	0.66 ± 0.02	8.02 ± 0.15	164.90 ± 5.46	64.89 ± 3.33^∗^
	25	0.66 ± 0.01	7.94 ± 0.09	151.60 ± 6.44	61.88 ± 4.07^∗^
	50	0.52 ± 0.03^∗^	8.08 ± 0.23	123.90 ± 5.13^∗^	46.47 ± 2.46^∗^
*N. hiiranensis*	VH	0.72 ± 0.01	10.60 ± 0.24	110.20 ± 2.97	112.00 ± 3.91
	5	0.72 ± 0.01	12.09 ± 0.36	103.50 ± 8.71	93.28 ± 2.90^∗^
	10	0.72 ± 0.01	12.09 ± 0.16	84.78 ± 7.32^∗^	87.92 ± 5.67^∗^
	25	0.75 ± 0.01	11.95 ± 0.57	80.71 ± 6.52^∗^	83.18 ± 4.46^∗^
	50	0.80 ± 0.03	9.80 ± 0.19	59.68 ± 11.64^∗^	60.32 ± 4.95^∗^
*N. konishii*	VH	0.72 ± 0.00	17.07 ± 0.32	94.47 ± 5.90	127.90 ± 8.11
	5	0.74 ± 0.01	14.99 ± 0.43^∗^	56.30 ± 7.77^∗^	132.50 ± 9.40
	10	0.74 ± 0.03	14.10 ± 0.60^∗^	42.55 ± 3.01^∗^	114.60 ± 5.03
	25	0.67 ± 0.01	12.19 ± 0.33^∗^	38.55 ± 4.37^∗^	120.70 ± 2.99
	50	0.35 ± 0.01^∗^	10.39 ± 0.38^∗^	27.30 ± 3.88^∗^	116.80 ± 5.19
*N. parvigemma*	VH	0.35 ± 0.02	9.30 ± 1.18	196.70 ± 14.12	243.80 ± 8.27
	5	0.32 ± 0.03	8.42 ± 0.25	178.40 ± 15.70	218.90 ± 7.82
	10	0.33 ± 0.01	8.29 ± 0.23	146.50 ± 4.94^∗^	189.70 ± 3.67^∗^
	25	0.30 ± 0.02	8.35 ± 0.26	140.60 ± 9.96^∗^	172.40 ± 4.48^∗^
	50	0.30 ± 0.02	8.35 ± 0.15	119.60 ± 5.20^∗^	157.00 ± 4.89^∗^
*N. sericea* var. *aurata*	VH	0.65 ± 0.03	21.21 ± 1.22	393.30 ± 16.52	147.80 ± 11.74
	5	0.66 ± 0.02	22.86 ± 0.43	314.30 ± 12.97^∗^	109.50 ± 3.70^∗^
	10	0.67 ± 0.02	23.98 ± 2.31	307.50 ± 11.61^∗^	87.00 ± 4.91^∗^
	25	0.67 ± 0.01	21.76 ± 0.95	215.60 ± 16.29^∗^	72.83 ± 3.16^∗^
	50	0.61 ± 0.07	20.26 ± 0.33	199.80 ± 26.79^∗^	69.50 ± 4.17^∗^
*N. villosa*	VH	0.47 ± 0.03	7.95 ± 0.54	87.60 ± 4.38	78.08 ± 2.72
	5	0.42 ± 0.01	8.28 ± 0.11	77.24 ± 7.08	81.61 ± 3.81
	10	0.44 ± 0.01	8.21 ± 0.65	61.96 ± 1.89^∗^	95.23 ± 2.68
	25	0.52 ± 0.02	8.21 ± 0.44	50.67 ± 8.33^∗^	90.93 ± 2.91
	50	0.55 ± 0.02	7.54 ± 0.28	29.95 ± 4.15^∗^	73.51 ± 3.18

Data were expressed as the mean ± SE of quadruplicate cultures. Results were representative of four independent experiments. ^∗^*p* < 0.05 was significant compared to the VH group.

**Table 3:** The effects of *N*. species root extracts on Th1/Th2 cytokine production by ConA-stimulated splenocytes.

Plants	Concentration (*μ*g/mL)	Cell viability	Th1 cytokines		Th2 cytokines
		MTT (O.D.)	IL-2 (ng/mL)	IFN-*γ* (ng/mL)	IL-4 (pg/mL)
	Control	0.06 ± 0.00	0.14 ± 0.12	Not detectable	Not detectable
*N. aciculata* var. *variabillima*	VH	0.40 ± 0.01	10.15 ± 0.71	66.21 ± 6.82	166.50 ± 5.30
	5	0.43 ± 0.02	9.31 ± 0.31	50.21 ± 7.96	171.00 ± 18.73
	10	0.41 ± 0.02	10.32 ± 0.22	40.42 ± 3.31^∗^	210.20 ± 18.04
	25	0.37 ± 0.03	8.02 ± 0.11^∗^	21.15 ± 2.55^∗^	141.20 ± 7.61
	50	0.29 ± 0.02^∗^	5.90 ± 0.31^∗^	8.379 ± 2.25^∗^	86.02 ± 6.78^∗^
*N. konishii*	VH	0.68 ± 0.00	12.91 ± 0.09	99.63 ± 8.09	102.40 ± 1.41
	5	0.69 ± 0.01	13.01 ± 0.13	64.55 ± 4.33^∗^	106.70 ± 4.83
	10	0.72 ± 0.01	13.21 ± 0.14	48.06 ± 6.32^∗^	107.60 ± 4.82
	25	0.71 ± 0.03	13.05 ± 0.10	25.46 ± 2.70^∗^	106.20 ± 7.55
	50	0.64 ± 0.01	12.45 ± 0.21	7.93 ± 1.34^∗^	102.00 ± 8.80

Data were expressed as the mean ± SE of quadruplicate cultures. Results were representative of four independent experiments. ^∗^*p* < 0.05 was significant compared to the VH group.
