# Supplementary material for: Immunomodulatory Effects of Taiwanese Neolitsea Species on Th1 and Th2 Functionality
Source: J Immunol Res. 2017 Jul 11;2017:3529859. doi: 10.1155/2017/3529859 (PMC5525079; doi:10.1155/2017/3529859)
Supplement: Supplementary file 1 — The information of supplementary materials are as follows: Supplemental Table 1. The Effects of N. species extracts on Th1/Th2 cytokine production by ConA-stimulated splenocytes. Supplemental Fig 1. The effects of selected Neolitsea species extracts on IL-12 secretions by ConA-stimulated splenocytes. ConA-stimulated splenocytes (5∗106cells/mL) were either left untreated (NA) or re-stimulated with ConA (5 µg/mL) in the absence or the presence of selected Neolitsea species (5-50 µg/mL) for 48 h. (A-C) The levels of IL-12 secretions in the supernatants were quantified by ELISA assay. Data were expressed as the mean ± SE of quadruplicate cultures. Results were representative of two independent experiments. ∗p<0.05 was significant compared to the VH group. [file 3529859.f1.docx]

| **Supplemental Table 1. The Effects of *N.* species extracts on Th1/Th2 cytokine production by ConA-stimulated splenocytes** | | | | | | | | | | | | | | |
| --- | --- | --- | --- | --- | --- | --- | --- | --- | --- | --- | --- | --- | --- | --- |
| Plants | Parts of plants | Concentration | Cell viability | | | Th1 cytokines | | | | | | Th2 cytokine | | |
|  |  | (μg/mL) | MTT (O.D.) | | | IL-2 (ng/mL) | | | IFN-γ (ng/ml) | | | IL-4 (pg/ml) | | |
| ***N. aciculata*** | Leaves | 0 | 0.66 | ± | 0.00 | 6.53 | ± | 0.08 | 119.30 | ± | 6.28 | 173.70 | ± | 8.07 |
|  |  | 10 | 0.68 | ± | 0.01 | 5.91 | ± | 0.10 | 121.30 | ± | 6.50 | 195.80 | ± | 29.09 |
|  | Stem | 0 | 0.38 | ± | 0.01 | 9.04 | ± | 0.18 | 93.82 | ± | 2.28 | 178.50 | ± | 3.55 |
|  |  | 10 | 0.45 | ± | 0.02 | 8.60 | ± | 0.07 | 77.04 | ± | 2.77* | 146.40 | ± | 4.32* |
|  | Root | 0 | 0.36 | ± | 0.01 | 7.12 | ± | 0.12 | 67.71 | ± | 0.32 | 150.00 | ± | 4.13 |
|  |  | 10 | 0.39 | ± | 0.01 | 7.36 | ± | 0.08 | 57.33 | ± | 2.29 | 137.30 | ± | 5.46 |
|  |  |  |  |  |  |  |  |  |  |  |  |  |  |  |
| ***N. aciculata* var. *variabillima*** | Leaves | 0 | 0.35 | ± | 0.01 | 13.15 | ± | 0.47 | 94.04 | ± | 2.37 | 310.30 | ± | 7.96 |
|  |  | 10 | 0.33 | ± | 0.01 | 11.97 | ± | 0.36 | 59.23 | ± | 5.86* | 300.10 | ± | 5.23 |
|  | Stem | 0 | 0.35 | ± | 0.01 | 12.16 | ± | 0.38 | 109.4 | ± | 6.07 | 256.10 | ± | 1.46 |
|  |  | 10 | 0.35 | ± | 0.01 | 13.28 | ± | 0.30 | 80.94 | ± | 5.52* | 216.90 | ± | 7.34* |
|  | Root | 0 | 0.40 | ± | 0.01 | 7.50 | ± | 0.14 | 63.64 | ± | 3.00 | 131.40 | ± | 6.57 |
|  |  | 10 | 0.41 | ± | 0.02 | 6.76 | ± | 0.18 | 40.55 | ± | 2.10* | 161.00 | ± | 1.86 |
|  |  |  |  |  |  |  |  |  |  |  |  |  |  |  |
| ***N. acuminatissima*** | Leaves | 0 | 0.71 | ± | 0.01 | 13.11 | ± | 0.70 | 108.90 | ± | 22.14 | 51.62 | ± | 4.05 |
|  |  | 10 | 0.68 | ± | 0.06 | 14.09 | ± | 1.11 | 70.09 | ± | 7.43* | 18.03 | ± | 8.28* |
|  | Stem | 0 | 0.71 | ± | 0.01 | 12.89 | ± | 1.18 | 417.00 | ± | 22.50 | 52.90 | ± | 4.91 |
|  |  | 10 | 0.65 | ± | 0.02 | 12.70 | ± | 0.88 | 146.10 | ± | 17.68* | 30.35 | ± | 1.84* |
|  | Root | 0 | 0.67 | ± | 0.06 | 11.19 | ± | 0.49 | 100.60 | ± | 10.63 | 49.52 | ± | 3.64 |
|  |  | 10 | 0.64 | ± | 0.03 | 11.16 | ± | 0.36 | 73.16 | ± | 4.06* | 34.35 | ± | 2.32* |
|  |  |  |  |  |  |  |  |  |  |  |  |  |  |  |
| ***N. buisanensis*** | Leaves | 0 | 0.66 | ± | 0.02 | 17.01 | ± | 0.53 | 78.90 | ± | 4.13 | 41.25 | ± | 6.07 |
|  |  | 10 | 0.66 | ± | 0.01 | 15.31 | ± | 0.30 | 74.40 | ± | 1.58 | 22.15 | ± | 1.25* |
|  | Stem | 0 | 0.61 | ± | 0.03 | 17.04 | ± | 0.52 | 147.20 | ± | 5.66 | 50.34 | ± | 1.13 |
|  |  | 10 | 0.76 | ± | 0.01 | 16.02 | ± | 0.44 | 102.00 | ± | 3.64* | 22.30 | ± | 2.62* |
|  | Root | 0 | 0.47 | ± | 0.01 | 4.91 | ± | 0.17 | 93.54 | ± | 2.47 | 83.41 | ± | 1.88 |
|  |  | 10 | 0.41 | ± | 0.02* | 4.33 | ± | 0.07* | 0.28 | ± | 0.21* | 43.75 | ± | 2.15* |
|  |  |  |  |  |  |  |  |  |  |  |  |  |  |  |
| ***N. daibuensis*** | Leaves | 0 | 0.60 | ± | 0.02 | 5.77 | ± | 0.29 | 157.30 | ± | 10.25 | 190.60 | ± | 18.56 |
|  |  | 10 | 0.69 | ± | 0.02 | 5.75 | ± | 0.21 | 164.90 | ± | 5.46 | 142.20 | ± | 4.72* |
|  | Stem | 0 | 0.30 | ± | 0.01 | 9.15 | ± | 0.24 | 156.60 | ± | 8.05 | 76.02 | ± | 1.48 |
|  |  | 10 | 0.31 | ± | 0.01 | 8.74 | ± | 0.18 | 167.30 | ± | 5.42 | 67.49 | ± | 2.29 |
|  | Root | 0 | 0.51 | ± | 0.02 | 4.37 | ± | 0.16 | 100.90 | ± | 2.58 | 32.41 | ± | 0.89 |
|  |  | 10 | 0.52 | ± | 0.01 | 4.73 | ± | 0.12 | 102.00 | ± | 10.11 | 21.61 | ± | 1.30* |
|  |  |  |  |  |  |  |  |  |  |  |  |  |  |  |
| ***N. hiiranensis*** | Leaves | 0 | 0.79 | ± | 0.01 | 10.64 | ± | 0.14 | 104.60 | ± | 8.82 | 115.40 | ± | 8.83 |
|  |  | 10 | 0.81 | ± | 0.01 | 12.07 | ± | 0.22 | 86.22 | ± | 2.78* | 90.12 | ± | 2.79* |
|  | Stem | 0 | 0.79 | ± | 0.01 | 12.53 | ± | 0.41 | 124.40 | ± | 18.08 | 33.80 | ± | 1.05 |
|  |  | 10 | 0.81 | ± | 0.02 | 12.54 | ± | 0.31 | 97.22 | ± | 7.43* | 23.88 | ± | 3.35* |
|  | Root | 0 | 0.72 | ± | 0.00 | 11.36 | ± | 0.27 | 133.90 | ± | 10.59 | 120.40 | ± | 17.45 |
|  |  | 10 | 0.66 | ± | 0.06 | 9.30 | ± | 0.19* | 14.25 | ± | 0.77* | 69.40 | ± | 11.22* |
|  |  |  |  |  |  |  |  |  |  |  |  |  |  |  |
| ***N. konishii*** | Leaves | 0 | 0.72 | ± | 0.00 | 17.18 | ± | 0.29 | 87.55 | ± | 8.08 | 141.20 | ± | 15.86 |
|  |  | 10 | 0.74 | ± | 0.03 | 14.72 | ± | 1.04* | 42.55 | ± | 3.01* | 116.60 | ± | 0.43 |
|  | Stem | 0 | 0.81 | ± | 0.04 | 13.57 | ± | 0.13 | 101.10 | ± | 3.97 | 119.40 | ± | 23.75 |
|  |  | 10 | 0.69 | ± | 0.04 | 13.22 | ± | 0.30 | 96.57 | ± | 5.84 | 105.50 | ± | 5.89 |
|  | Root | 0 | 0.68 | ± | 0.00 | 12.91 | ± | 0.09 | 99.63 | ± | 8.09 | 102.40 | ± | 1.41 |
|  |  | 10 | 0.72 | ± | 0.01 | 13.21 | ± | 0.14 | 61.31 | ± | 0.78* | 107.60 | ± | 4.82 |
|  |  |  |  |  |  |  |  |  |  |  |  |  |  |  |
| ***N. parvigemma*** | Leaves | 0 | 1.05 | ± | 0.02 | 9.55 | ± | 0.26 | 196.70 | ± | 14.12 | 243.80 | ± | 8.27 |
|  |  | 10 | 1.04 | ± | 0.03 | 8.29 | ± | 0.23 | 148.50 | ± | 5.18* | 189.70 | ± | 3.67* |
|  | Stem | 0 | 1.05 | ± | 0.02 | 9.62 | ± | 0.19 | 200.70 | ± | 15.56 | 219.40 | ± | 6.58 |
|  |  | 10 | 1.03 | ± | 0.03 | 8.20 | ± | 0.30 | 124.40 | ± | 1.82* | 148.10 | ± | 3.61* |
|  | Root | 0 | 1.08 | ± | 0.02 | 10.14 | ± | 0.73 | 180.20 | ± | 14.81 | 184.10 | ± | 10.22 |
|  |  | 10 | 1.01 | ± | 0.04 | 11.37 | ± | 0.17 | 104.40 | ± | 11.00* | 162.90 | ± | 5.80 |
|  |  |  |  |  |  |  |  |  |  |  |  |  |  |  |
| ***N. sericea var. aurata*** | Leaves | 0 | 0.65 | ± | 0.03 | 19.88 | ± | 0.43 | 358.50 | ± | 22.62 | 264.50 | ± | 30.78 |
|  |  | 10 | 0.67 | ± | 0.02 | 20.16 | ± | 1.15 | 261.80 | ± | 8.33* | 194.80 | ± | 12.55* |
|  | Stem | 0 | 0.65 | ± | 0.03 | 22.66 | ± | 0.40 | 362.90 | ± | 7.38 | 217.80 | ± | 15.14 |
|  |  | 10 | 0.60 | ± | 0.02 | 21.21 | ± | 1.29 | 265.70 | ± | 16.47* | 160.90 | ± | 2.37* |
|  |  |  |  |  |  |  |  |  |  |  |  |  |  |  |
| ***N. villosa*** | Leaves | 0 | 0.48 | ± | 0.02 | 7.95 | ± | 0.54 | 85.09 | ± | 6.29 | 170.00 | ± | 3.27 |
|  |  | 10 | 0.48 | ± | 0.02 | 8.21 | ± | 0.65 | 59.96 | ± | 1.59* | 184.20 | ± | 5.60 |
|  | Stem | 0 | 0.48 | ± | 0.02 | 5.96 | ± | 0.42 | 59.77 | ± | 8.59 | 134.50 | ± | 7.04 |
|  |  | 10 | 0.42 | ± | 0.00 | 6.39 | ± | 0.63 | 48.40 | ± | 2.78* | 120.90 | ± | 2.45 |
|  | Root | 0 | 0.40 | ± | 0.01 | 17.67 | ± | 0.50 | 55.75 | ± | 1.95 | 161.20 | ± | 8.78 |
|  |  | 10 | 0.41 | ± | 0.02 | 16.17 | ± | 0.40 | 9.95 | ± | 0.51* | 184.20 | ± | 5.60 |

Data were expressed as the mean ± SE of quadruplicate cultures. Results were representative of four independent experiments. **p*<0.05 was significant compared to the control group.
