# Supplementary material for: Immunomodulatory Effects of Taiwanese Neolitsea Species on Th1 and Th2 Functionality
Source: J Immunol Res. 2017 Jul 11;2017:3529859. doi: 10.1155/2017/3529859 (PMC5525079; doi:10.1155/2017/3529859)
Supplement: Supplementary file 2 [file 3529859.f2.docx]

**
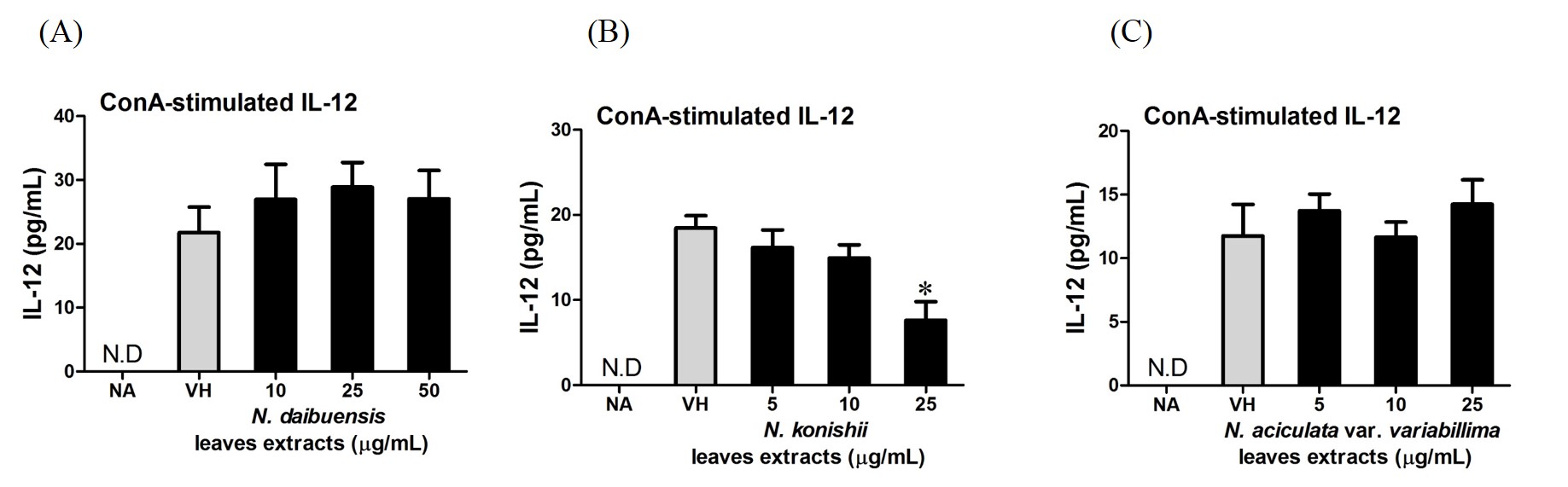
**

**Supplemental Fig 1. The effects of selected *Neolitsea* species extracts on IL-12 secretions by ConA-stimulated splenocytes.** ConA-stimulated splenocytes (5*10^6^cells/mL) were either left untreated (NA) or re-stimulated with ConA (5 μg/mL) in the absence or the presence of selected *Neolitsea* species (5-50 μg/mL) for 48 h. (A-C) The levels of IL-12 secretions in the supernatants were quantified by ELISA assay. Data were expressed as the mean ± SE of quadruplicate cultures. Results were representative of two independent experiments. **p*<0.05 was significant compared to the VH group.
